# Racial inequality in access to breast reconstruction surgery in the Brazilian Unified Health System: an interrupted time series study, Brazil, 2010-2024

**DOI:** 10.1590/S2237-96222026v35e20250496.en

**Published:** 2026-07-24

**Authors:** Alana Ramos da Silva, Fernando Antonio Slaibe Postali

**Affiliations:** 1 Universidade de São Paulo, Faculdade de Economia, Administração e Contabilidade, São Paulo, SP, Brazil Universidade de São Paulo Faculdade de Economia Administração e Contabilidade São Paulo SP Brazil

**Keywords:** Breast Reconstruction, Racial Inequality in Health, Breast Cancer, Brazilian Unified Health System, Mastectomy., Reconstrucción Mamaria, Desigualdad Racial en la Salud, Cáncer de Mama, Sistema Único de Salud de Brasil, Mastectomía.

## Abstract

**Objective:**

To estimate the differential in access to breast reconstruction surgery in the Brazilian Unified Health System according to race/skin color nationwide and in the country's regions.

**Methods:**

This was an observational study of interrupted time series, which used monthly microdata from the Hospital Information System for the period 2010-2024. The differentials in the proportion of breast reconstruction surgeries according to race/skin color were estimated before and after the implementation of Ordinance No. 127/2023, with respective 95% confidence intervals (95%CI).

**Results:**

We identified 565,234 surgeries, of which 404,944 (71.6%) were mastectomies and 160,290 (28.4%) were breast reconstructions. The proportion of reconstructions was, on average, 12.9 percentage points (95%CI -16.8; -8.9) lower for Black women compared to White and Asian women. After the Ordinance No. 127/2023 intervention, the differential increased to 28.6 percentage points (95%CI -44.1; -13.0). The largest differentials were observed in the Northern region, with 15.4 percentage points (95%CI -20.9; -10.8), and in the Northeast, with 11.7 percentage points (95%CI -15.8; -7.5).

**Conclusion:**

There is a significant differential in access to breast reconstruction surgery according to race/skin color, and the effects are heterogeneous across the Brazilian regions.

## Resumen

**Objetivo:**

Estimar la diferencia en el acceso a la cirugía de reconstrucción mamaria en el Sistema Único de Salud de Brasil según la raza/color de piel en Brasil y sus regiones.

**Métodos:**

Estudio observacional de series temporales interrumpidas, que utilizó microdatos mensuales del Sistema de Información Hospitalaria para el periodo 2010-2024. Se estimaron las diferencias en la proporción de cirugías de reconstrucción mamaria según la raza/color de piel antes y después de la implementación de la Ordenanza N° 127/2023, con sus respectivos intervalos de confianza del 95% (IC95%).

**Resultados:**

Se identificaron 565.234 cirugías, de las cuales 404.944 (71,6 %) fueron mastectomías y 160.290 (28,4 %) reconstrucciones mamarias. La proporción de reconstrucciones fue, en promedio, 12,9 puntos porcentuales (IC95 % -16,8; -8,9) menor para las mujeres negras en comparación con las mujeres blancas y asiáticas. Tras la entrada en vigor de la Ordenanza n.º 127/2023, la diferencia aumentó a 28,6 puntos porcentuales (IC95% -44,1; -13,0). Las mayores diferencias se observaron en la región Norte, con 15,4 puntos porcentuales (IC95% -20,9; -10,8), y en el Noreste, con 11,7 puntos porcentuales (IC95% -15,8; -7,5).

**Conclusión:**

Existe una diferencia significativa en el acceso a la cirugía de reconstrucción mamaria según la raza/color de piel, y los efectos son heterogéneos entre las regiones.

**Ethical aspects**

This study used publicly available and anonymized databases.

## Introduction

Breast cancer has the highest mortality rate and the second highest incidence rate among cancers that most commonly affect Brazilian women. In 2024, 73,000 estimated new cases were expected in the country. The most appropriate treatment depends on the stage and type of tumor[xref ref-type="bibr" rid="r1"]^1^[/xref].

The Brazilian Unified Health System (*Sistema Único de Saúde*, SUS) offers free breast cancer surgery, chemotherapy and radiotherapy^1^. Breast surgery, called mastectomy, is one of the main treatment modalities. Radical mastectomy, with total removal of the breast, was once considered the standard treatment, based on the belief that increasing the extent of the surgery would produce better results^2^. Currently, breast-conserving techniques are seen as an effective treatment option^3^^,^^4^, allowing for less invasive and painful treatments for health service users.

Breast reconstruction is performed after conservative surgery for local breast repairs and symmetrization^5^ and aims to restore patients' self-esteem and psychological well-being^6^^,^^7^. It is therefore seen as essential for the physical and emotional recovery of women undergoing treatment for breast cancer^8^.

Despite this, access to breast reconstruction has remained below the number of mastectomies performed^9^. This causes considerable suffering for women who are unable to complete treatment. In low- and middle-income countries, reconstruction rates are far from ideal, as they do not reach the 50% mark^10^. In 2023, the Brazilian Ministry of Health conducted an analysis that indicated an increase in access to this procedure from 21% in 2010 to approximately 65% ​​in 2021^9^. However, it was estimated that less than 30% of patients who underwent mastectomy had reconstruction through the SUS between 2015 and 2021^11^^-^^12^.

With a view to patients' quality of life, Law No. 12802/2013 was brought into force by the SUS, which guarantees access to breast reconstruction immediately after tumor removal or as soon as the necessary clinical conditions exist^13^, and created an exceptional strategy to expand access in 2023^14^. Ordinance No. 127 was enacted in February 2023 and included in the SUS health systems in June of the same year. The exceptional strategy provides that hospitals already accredited to provide SUS oncology services may perform post-mastectomy breast reconstruction, establishing a new procedure within the SUS^14^.

The main cause of low access to breast reconstruction is unequal access to healthcare. The literature indicates that race/skin color, place of residence and social class on their own do not affect health outcomes. In fact, these factors are related to overlapping processes that generate inequities, such as racism and other forms of discrimination and oppression^15^. At different times, disparities have been noted in some breast cancer indicators: Black women have less access to screening^16^, longer waiting times to start treatment^17^, higher incidence of diagnoses at advanced stages^18^, and higher risk of mortality^19^.

The objective of this study was to estimate the differential in access to breast reconstruction according to race/skin color between January 2010 and December 2024 in Brazil as a whole and in the country's regions. Analyses such as these allow identification of the women and locations that most need public policies aimed at promoting more equitable access to complete breast cancer treatment.

## Methods

Design 

This is an observational study with interrupted time series analysis using panel data, based on monthly records of surgeries performed between January 2010 and December 2024 held on the SUS Hospital Information System. These microdata are publicly accessible and available on the SUS Department of Information Technology platform^20^.

Setting

Monthly rates were calculated and the aforementioned method was applied in order to assess the effects of Ordinance No. 127, enacted in February 2023 and brought into effect in June of the same year. The objective was to estimate differentials according to race/skin color in access to breast reconstruction surgery after mastectomy. The approach allowed for analysis of differences in the trend (slope) of monthly rates between the two groups of women (Black/mixed-race vs. White/Asian) before and after the intervention brought about by Ordinance No. 127.

Participants

The study investigated mastectomy and breast reconstruction surgeries performed on women between January 2010 and December 2024. The unit of analysis was the monthly rates by racial group. The number of surgeries was obtained based on the proc_id variable. The mastectomy codes were selected as per Silva et al.^21^, while the codes for breast reconstruction procedures were obtained based on Almeida et al.^11^.

Variables

We analyzed race/skin color and region of residence markers. The raca_cor (race_color) variable identified the SUS service user's race/skin color as established by the Brazilian Institute of Geography and Statistics, whereby 1 indicates White race/skin color, 2 Black, 3 mixed-race, 4 Asian, 5 Indigenous, 99 unknown or "no information". Race/skin color data coded as 99 was removed from the study. In total, 120,492 surgeries did not have information on race/skin color, including 88,170 mastectomies and 32,322 breast reconstruction surgeries. Indigenous women were excluded from the analysis due to their low representation in the sample (137 mastectomies and 23 reconstructions).

Information on race/skin color became mandatory on the Hospital Information System in November 2022, making the "no information" option for race/skin color unavailable during data entry. The data shows that during the two-year period 2023 and 2024, there were no data entries with missing race/skin color information. This information is recorded by healthcare professionals and is therefore not self-declared.

Our analysis focused on two groups of women: those with codes 2 and 3 in the database, that is, Black and mixed-race (both classified as Black in this study); and White and Asian women, i.e. codes 1 and 4, respectively. The Black population includes people who self-identify as both Black and mixed-race. Although it corresponds to a wide range of phenotypes and diverse genomic ancestries, this political and social categorization is frequently adopted in Brazil.

Region of residence was obtained via the *munic_res* variable, which indicates the service user's municipality code, whereby when the first character is 1 it corresponds to the Northern region, 2 to the Northeast, 3 to the Southeast, 4 to the South and 5 to the Midwest.

Data source/measurement 

The database was extracted from the Hospital Information System in March 2025 and consists of two files available for download on the File Transfer Platform^20^. In this study, the reduced database was used, from which it was possible to obtain information on surgeries and the criteria regarding the race/skin color and region of residence of the health service users. The files are monthly and organized according to the country's federative units.

The breast reconstruction indicator (dependent variable) was calculated as the percentage of reconstructions in relation to mastectomies, that is, where *Rate*_*it*_ is the relationship between breast reconstruction surgeries and mastectomies performed in group *i* (Black or White and Asian women) during month *t*. *Reconstructions*_*it*_ and *Masctectomies* indicate the total number of group *i* reconstructions and mastectomies during month *t*, respectively.

$$Rate_{it}=\frac{Reconstructions_{it}}{Mastectomies_{it}}\cdot 100\left(1\right)$$

Service users have the right to undergo reconstruction immediately after tumor removal, if the necessary clinical conditions are met, or as soon as the requirements are met^13^. There are three possibilities: reconstruction is indicated immediately after mastectomy; reconstruction is not indicated; or, firstly, the patient must undergo complementary treatment with chemotherapy/radiotherapy and then breast reconstruction^5^. In general, the decision on the best treatment is personalized according to the specificity of the tumor, age group and daily activities. In addition, the decision is shared between the mastologist, the plastic surgeon, the oncologist and the patient^5^.

The microdata do not include user identification to track when each participant underwent reconstruction. Rates were calculated monthly for each of the two groups of women. This implies that this study enriched the literature by analyzing the differential according to race/skin color using monthly rates.

The annual number of breast reconstructions did not reflect the number of women undergoing mastectomy after breast cancer diagnosis^9^. Expression (1) described the monthly capacity of the SUS to offer complete treatment for the disease.

Bias 

The exclusion of records lacking information on race/skin color affected a substantial portion of the sample. Furthermore, the COVID-19 pandemic reduced elective surgeries between 2020 and 2021.

Study size 

After excluding records lacking race/skin color information and excluding information on Indigenous women, the final sample analyzed consisted of 160,290 reconstructions and 404,944 mastectomies. Because these are population-based administrative data, no sample size calculation was performed.

Statistical methods 

The interrupted time series with panel data method was described in Linden^22^. The Ordinance No. 127/2023 intervention was explored to obtain the differential according to race/skin color in the relationship between breast reconstruction surgeries and mastectomies in the SUS. The Ordinance established an exceptional strategy to expand access to reconstruction^14^ and was included in the SUS Table of Procedures, Medicines and Orthoses, Prostheses and Mobility Aids Management System, popularly known as the SUS Table, in June 2023.

The identification hypothesis was that the change in the level or trend of the rates would be the same for the control group (White and Asian) and for the treatment group (Black) if the intervention had not occurred^22^. The intervention caused by the COVID-19 pandemic is not suitable for this analysis, as many oncology surgeries were interrupted during that period. Consequently, this reduced the differential between the groups. The effect of the pandemic is always positive when comparing the rates with the substantial reduction that occurred from March 2020 onwards.

The model was estimated using ordinary least squares, as follows:

*Rate*_*it*_ *= ß*_*0*_ *+ ß*_*1*_ *Time*_*it*_ *+ß*_*2*_ *Intervention*_*it*_ *+ ß*_*3*_ *Time*_*it*_ *· Intervention*_*it*_ *+ ß*_*4*_ *Black women*_*i*_ *+ ß*_*5*_ *Black women*_*i*_ *· Time*_*it*_ *+ ß*_*6*_ *Black women*_*i*_ *· Intervention*_*it*_ *+ ß*_*7*_ *Black women*_*i*_ *· Intervention*_*it*_ *· Time*_*it*_ *+ ε*_*it*_ (2)

where:

*Rate*_*it*_ is the dependent variable calculated in (1);

*Time*_*it*_ is a continuous variable varying between 1 and 180, representing January 2010 to December 2024 for each i group;

*Intervention*_*it*_ is a binary variable that receives 1 with effect from June 2023;

*Time*_*it*_ *· Intervention*_*it*_ is a continuous variable that indicates the time elapsed since the intervention, that is, in July 2023 it is 1, in August 2023 is it 2 and so on;

*Black women*_*i*_ is a binary variable that indicates 1 for the Black women's group and 0 for the White and Asian women's group;

*Black women*_*i*_ *· Time*_*it*_ is the time elapsed since the beginning of the study for the Black women's group;

*Black women*_*i*_ *· Intervention*_*it*_ is a binary variable that receives 1 for the intervention for Black women;

*Black women*_*i*_ *· Intervention*_*it*_ *· Time*_*it*_ indicates the time with effect from the intervention for Black women; and

*ε*_*it*_ is the error term. 

Equation (2) tested comparability between groups. *ß*_*4*_ and *ß*_*5*_ indicate whether the groups are balanced in both the level and trajectory of the outcome variable in the pre-intervention period^22^. The *ß*_*0*_ to *ß*_*3*_ coefficients measure the effects for White and Asian women, while the *ß*_*4*_ to *ß*_*6*_ parameters measure the effects for Black women. Therefore, and are the parameters of interest and estimate the differential between the two groups of women before and after the intervention in June 2023, respectively. Specification (2) allows heterogeneous effects to be captured, adjusting for the different trajectories and levels of the dependent variable before and after the intervention^22^. Furthermore, it is possible to verify the effectiveness of the Ordinance in expanding access to breast reconstructions.

The coefficients of equation (2) were estimated using R statistical software. The standard errors are heteroscedasticity-robust, calculated from the White estimator, as implemented by the sandwich package. We used 95% confidence intervals.

## Results

The total sample consisted of 565,234 surgeries performed on women, of which 71.6% (404,494) were mastectomies and 28.4% (160,290) were breast reconstructions. The surgical procedures used to measure the dependent variable of the model were described (Table 1). In the South, 91.2% of reconstructions were performed on White women, and 59.6% in the Southeast; in the North, 81.6% were performed on mixed-race women, 74.2% in the Northeast and 61.6% in the Midwest (Table 2).

Between 2010 and 2024, the proportion of reconstructions relative to mastectomies was higher among White and Asian women compared to Black women in all years analyzed (Figures 1 and 2). In 2010, the proportions were 26.6% among White and Asian women and 21.1% among Black women, a difference of 5.5 percentage points. In 2020, due to the interruption of elective procedures, a reduction in proportions was observed in both groups, with values ​​of 38.0% among White and Asian women and 34.4% among Black women, resulting in a difference of 3.6 percentage points. After this period, the proportions increased again, reaching 69.9% and 53.9%, respectively, in 2024, maintaining a difference of 16.0 percentage points between the groups (Figures 1 and 2).

**Table 1:** Code, description, number (n) and percentage (%) of mastectomies and breast reconstructions performed by the Brazilian Unified Health System. Brazil, 2010-2024

Code	Description	n (%)
	Mastectomies	404,944 (100.0)
0416120032	Simple mastectomy in oncology	14,554 (3.6)
0410010111	Setorectomy/quadrantectomy	179,537 (44.3)
0416120024	Radical mastectomy with axillary lymphadenectomy in oncology	87,855 (21.7)
0410010120	Setorectomy/quadrantectomy with lymph node dissection	23,014 (5.7)
0416120059	Breast segmentectomy in oncology	77,870 (19.2)
0410010065	Simple mastectomy	9,747 (2.4)
0410010057	Radical mastectomy with lymph node dissection	12,367 (3.1)
	Breast reconstructions	160,290 (100.0)
0410010090	Post-mastectomy reconstructive breast surgery with prosthesis implant	17,527 (10.9)
0410010073	Non-cosmetic female breast reconstruction	79,646 (49.7)
0416080081	Reconstruction with myocutaneous flap	60,658 (37.8)
0410010219	Total post-mastectomy breast reconstruction (procedure created by Ordinance No. 127/2023)	2,459 (1.5)

**Table 2:** Absolute (n) and relative (%) frequencies of mastectomy and breast reconstruction surgeries performed by region of residence and race/skin color. Brazil, 2010-2024

Surgery/women	Brazil n (%)	North n (%)	Northeast n (%)	Southeast n (%)	South n (%)	Midwest n (%)
Mastectomies	404,944	22,092	98.181	189.267	74,989	20,415
White	190,251 (47.0)	1,883 (8.5)	11,867 (12.1)	104,420 (55.2)	66,622 (88.8)	5,459 (26.7)
Black	25,015 (6.2)	413 (1.9)	5,998 (6.1)	15,147 (8.0)	2,693 (3.6)	764 (3.7)
Mixed-race	181,991 (44.9)	19,050 (86.2)	76,861 (78.3)	66,956 (35.4)	5,269 (7.0)	13,855 (67.9)
Asian	7,687 (1.9)	746 (3.4)	3,455 (3.5)	2,744 (1.4)	405 (0.5)	337 (1.7)
Reconstruction	160,290	3,636	41,715	77,041	30,845	7,053
White	84,051 (52.4)	503 (13.8)	7,118 (17.1)	45,881 (59.6)	28,130 (91.2)	2,419 (34.3)
Black	8,346 (5.2)	72 (2.0)	2,532 (6.1)	4,780 (6.2)	788 (2.6)	174 (2.5)
Mixed-race	65,702 (41.0)	2,967 (81.6)	30,972 (74.2)	25,677 (33.3)	1,740 (5.6)	4,346 (61.6)
Asian	2,191 (1.4)	94 (2.6)	1,093 (2.6)	703 (0.9)	187 (0.6)	114 (1.6)

**Figure 1:**
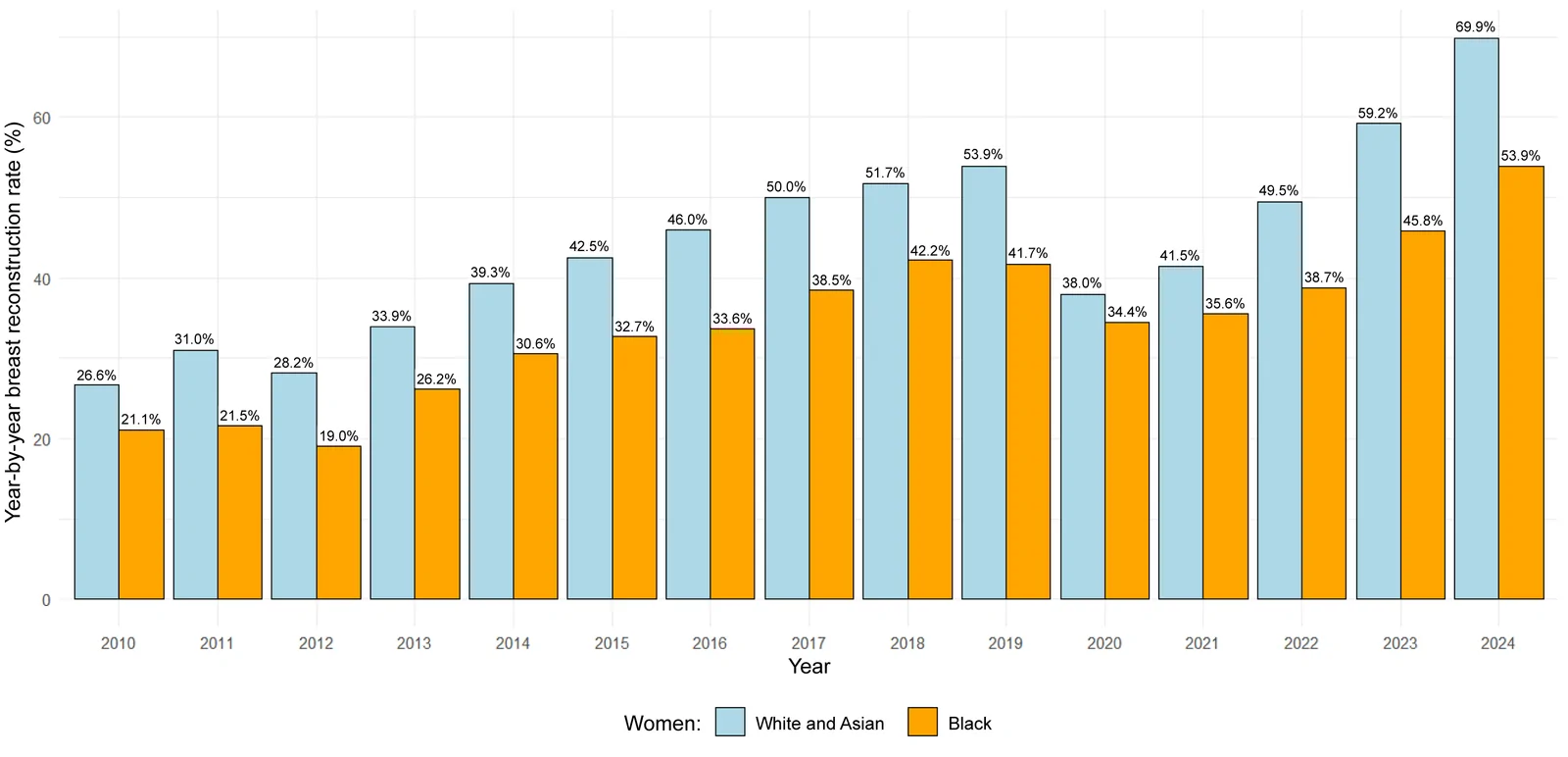
Year-by-year comparison of breast reconstruction rates per group of women. Brazil, 2010-2024

**Figure 2:**
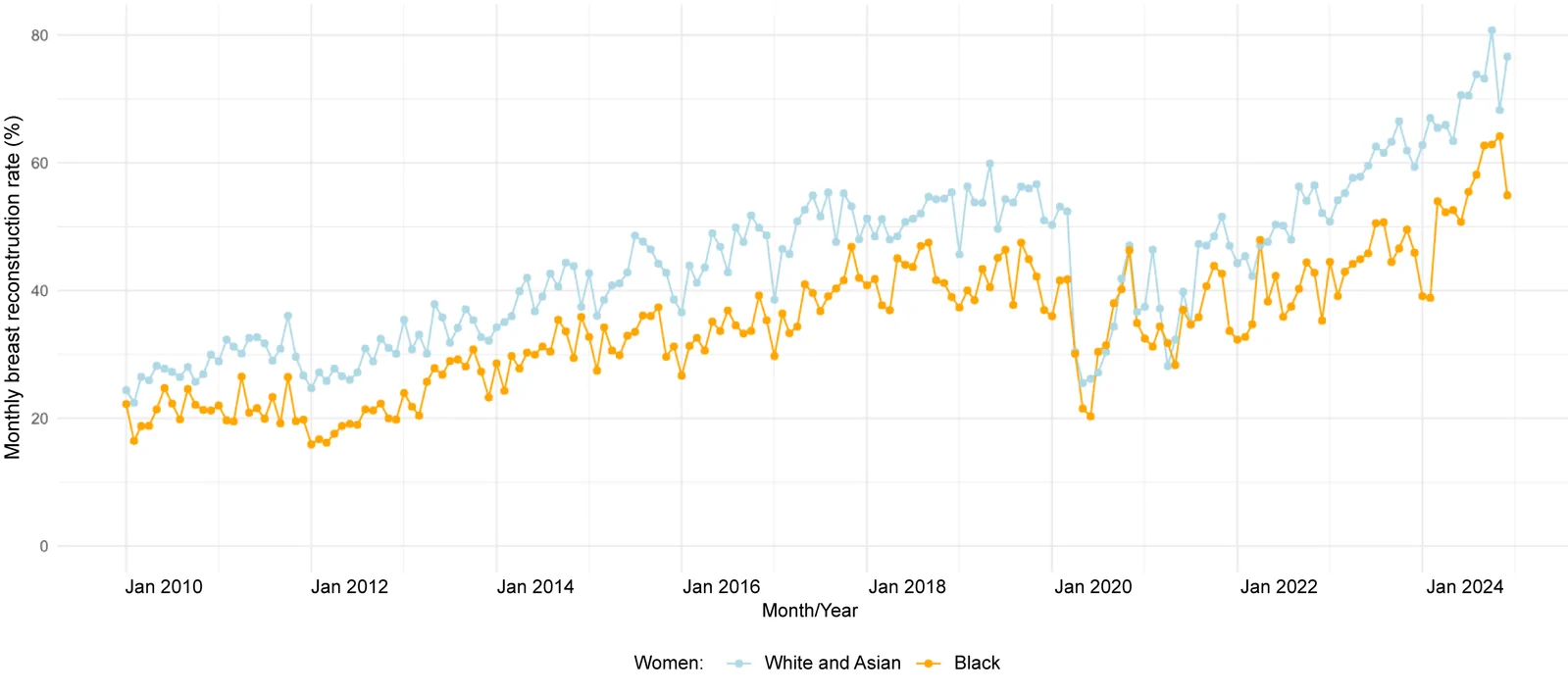
Monthly series of breast reconstruction rates per group of women. Brazil, 2010-2024

The monthly rates enabled equation (2) to be estimated for Brazil and for macro-regions of residence (North, Northeast, Southeast, South and Midwest) using filters in the database. In some months, the monthly rates showed proportions greater than 100% or could not be calculated in periods when no reconstructive surgeries were performed. In the Northern region, 20 observations were eliminated from the sample, seven months had rates above 100%, and no reconstructive surgeries occurred in thirteen of the months. These problems were observed in greater numbers between 2010 and 2014. The regions with the most complete information were the Southeast and South, with 360 and 359 observations, respectively (Table 3).

The significant coefficients for the *Black women*_*i*_ and *Black women*_*i*_ *· Intervention*_*it*_ variables of equation (2) indicated the existence of a racial/skin color differential in access to breast reconstruction in the SUS (Table 3). The *ß*_*4*_ coefficient of the *Black women*_*i*_ variable represented the group of Black women in equation (2) prior to the intervention and presented a negative and significant value in the estimates, except for the Midwest (Table 3). This indicated that Black women had lower rates of breast reconstruction surgery and mastectomies compared to the group of White and Asian women. 

For Brazil as a whole, the difference between the two groups was 12.9 percentage points (95% confidence interval, 95%CI -16.8; -8.9) in favor of White and Asian women in the pre-intervention period (Table 3). Regionally, the differential was 15.4 percentage points (95%CI -20.9; -10.8) in the North, 11.7 percentage points (95%CI -15.8; -7.5) in the Northeast, 8.0 percentage points (95%CI -11.0; -5.0) in the Southeast, and 6.9 percentage points (95%CI -10.8; -3.0) in the South. The models for the Northeast and Southern regions obtained the highest coefficients of determination, while those for the North and Midwest obtained the lowest (Table 3).

**Table 3:** Absolute difference (in percentage points) in breast reconstruction rates between Black women and White/Asian women before and after the Ordinance No. 127/2023 intervention, 95% confidence intervals (95%CI) and monthly observations by region of residence. Brazil, 2010-2024

Region	Before intervention (95%CI)	After intervention (95%CI)	Months
Brazil	-12.9 (-16.8; -8.9)	-28.6 (-44.1; -13.0)	351
North	-15.4 (-20.9; -10.8)	1.4 (-13.1; 16.0)	340
Northeast	-11.7 (-15.8; -7.5)	-21.0 (-46.3; 4.5)	340
Southeast	-8.0 (-11.0; -5.0)	-0.4 (-10.6; 9.7)	360
South	-6.9 (-10.8; -3.0)	-10.9 (-24.4; 2.5)	359
Midwest	1.8 (-3.8; 7.4)	-8.7 (-28.1; 10.7)	352

In the post-intervention estimates, the *ß*_*6*_ coefficient of the *Black women*_*i*_ *· Intervention*_*it*_ iteration of equation (2) was negative and highly significant only for Brazil as a whole. The differential increased to 28.6 percentage points (95%CI -44.1; -13.0) in favor of White and Asian women (Table 3).

## Discussion

These results indicated a racial/skin color differential in the relationship between breast reconstruction and mastectomy between January 2010 and December 2024. Rates were higher for White and Asian women compared to Black women. In Brazil, prior to the intervention made by Ordinance No. 127/2023, the differential was 12.9 percentage points, increasing to 28.6 percentage points from June 2023 onwards. Furthermore, the effects were heterogeneous across regions. The largest differentials were observed in the North and Northeast. Only in the Midwest region was the coefficient indicating the differential not significant.

The number of reconstructions did not match the number of mastectomies^9^^-^^12^. Using different annual timeframes, the proportions of reconstructions to mastectomies performed per year were verified^9^^-^^12^. In this way, this study contributed to the literature by analyzing the influence of race/skin color and by verifying regional heterogeneity using the time series method.

The limitations of this study are acknowledged. Firstly, the results were contingent on the accuracy and quality of the data. It was not possible to guarantee that there were no errors in information recording or information omission. Information on race/skin color became mandatory in the Hospital Information System records from November 2022. Therefore, the analyses may present some distortions. Secondly, it was not possible to control for the effects of the COVID-19 pandemic on the estimates. A reduction in oncology surgeries was observed during the most restrictive period of the pandemic^23^, so any comparison after the pandemic period will have a positive effect. Finally, the study's evidence was not based on a theoretical model that explains the relationships between all the possible factors involved.

Studies like this emphasize the importance of databases with complete information on race/skin color, with the aim of designing policies to reduce health inequalities. The results showed that the increase in reconstructions over the years did not ensure a reduction in access differences. This indicated the need for equitable public policies to expand access to reconstructive surgery.

Breast reconstruction is essential for the physical and emotional recovery of women undergoing cancer treatment^8^. The annual number of breast reconstructions has not reflected the increase in women diagnosed with breast cancer undergoing mastectomy^9^. Therefore, initiatives such as Ordinance No. 127/2023, which allowed referral hospitals to be able to perform post-mastectomy breast reconstruction^14^, are important to expand access to complete treatment^9^ and the well-being of service users.

These findings revealed the racial/skin color differential in access to breast reconstruction and are consistent with the literature. Many studies on breast cancer highlight the health inequalities faced by Black women. They have less access to screening^16^^,^^24^, present more advanced staging^17^ and higher risk of mortality^19^. The interactions of these multiple factors may influence differences in access to reconstruction, since more severe cases of cancer may present more contraindications for the procedure. Such adverse outcomes are also observed in socioeconomic indicators. Black women participate less in the labor market, are more affected by poverty and are less likely to complete higher education^25^.

Regional differences also stood out. The North and Northeast regions showed the greatest differences in access to reconstruction and were the areas with the highest proportion of Black and mixed-race people (79% and 73%, respectively)^26^. Despite progress in recent decades, inequalities persist. In the Northern region, breast cancer is the second most common cancer, after cervical cancer^27^, and has low screening rates^28^, longer waiting times for breast cancer surgery^29^ and limited access to cancer diagnosis and treatment due to the region's vast territorial dimensions^30^.

The concept of intersectionality is useful for explaining these inequalities, as it refers to the understanding that characteristics such as race/skin color, social class, gender and place of residence shape and reinforce complex social inequalities^15^. Therefore, there is a race/skin color differential in the monthly rates between breast reconstruction surgeries and mastectomies, and the effect is heterogeneous across the Brazilian regions. This result stems from differences in many social indicators that affect access to complete breast cancer treatment.

## Data Availability

The database used in this research is available at: https://doi.org/10.48331/scielodata.NPPFOF.
