# Supplementary material for: Racial inequality in access to breast reconstruction surgery in the Brazilian Unified Health System: an interrupted time series study, Brazil, 2010-2024
Source: Epidemiol Serv Saude. 2026 Jul 24;35:e20250496. doi: 10.1590/S2237-96222026v35e20250496.en (PMC13686374; doi:10.1590/S2237-96222026v35e20250496.en)
Supplement: Translation

# Translation: Desigualdade racial no acesso à cirurgia de reconstrução mamária no Sistema Único de Saúde: estudo de série temporal interrompida, Brasil, 2010-2024

**DOI:** 10.1590/S2237-96222026v35e20250496.pt

**Authors:** Alana Ramos da Silva, Fernando Antonio Slaibe Postali

**Affiliations:** 1 Universidade de São Paulo, Faculdade de Economia, Administração e Contabilidade, São Paulo, SP, Brasil Universidade de São Paulo Faculdade de Economia Administração e Contabilidade São Paulo SP Brasil

**Keywords:** Reconstrução Mamária, Desigualdade Racial em Saúde, Câncer de Mama, Sistema Único de Saúde, Mastectomia

## Abstract

**Objetivo:**

Estimar o diferencial no acesso à cirurgia de reconstrução mamária no Sistema Único de Saúde conforme raça/cor da pele no Brasil e regiões.

**Métodos:**

Estudo observacional de séries temporais interrompidas, o qual utilizou microdados mensais do Sistema de Informações Hospitalares no período 2010-2024. Estimaram-se os diferenciais na proporção de cirurgias de reconstrução mamária segundo raça/cor da pele, antes e após a implementação da Portaria nº 127/2023, com respectivos intervalos de confiança de 95% (IC95%).

**Resultados:**

Foram identificadas 565.234 cirurgias, sendo 404.944 (71,6%) mastectomias e 160.290 (28,4%) reconstruções mamárias. A proporção de reconstruções é, em média, 12,9 pontos percentuais (IC95% -16,8; -8,9) menor para mulheres negras em comparação com mulheres brancas e amarelas. Após a intervenção da Portaria nº 127/2023, o diferencial aumentou para 28,6 pontos percentuais (IC95% -44,1; -13,0). Os maiores diferenciais foram observados no Norte com 15,4 pontos percentuais (IC95% -20,9; -10,8) e no Nordeste com 11,7 pontos percentuais (IC95% -15,8; -7,5).

**Conclusão:**

Existe diferencial significativo no acesso à cirurgia de reconstrução mamária conforme raça/cor da pele, e os efeitos são heterogêneos entre as regiões.

**Aspectos éticos**

Este estudo utilizou banco de dados de domínio público e anonimizados.

## Introdução 

O câncer de mama apresenta a maior mortalidade e a segunda maior incidência entre os cânceres que mais acometem as mulheres brasileiras. Em 2024, foram estimados 73 mil novos casos no país. O tratamento mais adequado depende do estadiamento e do tipo do tumor^1^.

O Sistema Único de Saúde (SUS) oferece gratuitamente tratamento cirúrgico, quimioterapia e radioterapia^1^. A cirurgia, denominada mastectomia, é uma das principais modalidades de tratamento. A mastectomia radical, com a remoção total da mama, já foi considerada o tratamento padrão, com base na crença de que aumentar a extensão da cirurgia produziria melhores resultados^2^. Atualmente, técnicas conservadoras da mama são avaliadas como uma opção de tratamento eficaz^3^^,^^4^, o que permite tratamentos menos evasivos e dolorosos às usuárias.

A reconstrução mamária é realizada após a cirurgia conservadora para reparos locais e simetrização das mamas^5^ e tem como função reestabelecer a autoestima e o bem-estar psicológico das usuárias^6^^,^^7^. É vista, portanto, como essencial para a recuperação física e emocional das mulheres em tratamento contra o câncer de mama^8^.

Apesar disso, o acesso ao procedimento permaneceu aquém do número de mastectomias realizadas^9^. Isso causa sofrimento considerável para as mulheres que não conseguem finalizar o tratamento. Em países de baixa e média renda, as taxas de reconstrução estão longe do ideal, visto que não atingem o patamar de 50%^10^. Em 2023, o Ministério da Saúde realizou uma análise que indicou o aumento do acesso a esse procedimento de 21% em 2010 para cerca de 65% em 2021^9^. No entanto, foi estimado que menos de 30% das usuárias submetidas à mastectomia realizaram a reconstrução pelo SUS entre 2015 e 2021^11^^-^^12^.

Tendo em vista a qualidade de vida das usuárias, o SUS estabeleceu a Lei nº 12.802/2013, que garante o acesso à reconstrução mamária imediatamente após a retirada do tumor ou assim que houver as condições clínicas necessárias^13^, e criou uma estratégia excepcional para ampliar o acesso em 2023^14^. A Portaria nº 127 foi promulgada em fevereiro de 2023 e incluída nos sistemas de saúde do SUS em junho do mesmo ano. A estratégia excepcional prevê que hospitais já habilitados em oncologia no SUS possam realizar a reconstrução mamária pós-mastectomia, estabelecendo um novo procedimento nesse Sistema^14^.

A principal causa do baixo acesso às reconstruções mamárias é a desigualdade de acesso à saúde. A literatura indica que a raça/cor da pele, o local de residência e a classe social não afetam, de forma isolada, os resultados em saúde. Na verdade, esses fatores estão relacionados a processos sobrepostos que geram iniquidades, tais como racismo e outras formas de discriminação e opressão^15^. Em diferentes períodos, foram apontadas disparidades em alguns indicadores do câncer de mama: mulheres negras possuem menor acesso ao rastreamento^16^, maior tempo de espera para iniciar o tratamento^17^, maior incidência de diagnósticos em estágios avançados^18^ e maior risco de mortalidade^19^.

Este estudo teve como objetivo estimar o diferencial de acesso à reconstrução mamária conforme raça/cor da pele entre janeiro de 2010 e dezembro de 2024 no Brasil e regiões. Análises como essas permitem identificar as mulheres e as localidades que mais precisam de políticas públicas, visando promover o acesso mais igualitário ao tratamento completo do câncer de mama. 

## Métodos

Delineamento 

Trata-se de estudo observacional com análise de séries temporais interrompidas com dados em painel, a partir de registros mensais de cirurgias realizadas entre janeiro de 2010 e dezembro de 2024 do Sistema de Informações Hospitalares do SUS. Esses microdados são de acesso público e estão disponíveis na plataforma do Departamento de Informática do Sistema Único de Saúde^20^. 

Contexto do estudo 

Foram calculadas taxas mensais e aplicou-se o método supracitado para avaliar os efeitos da Portaria nº 127, promulgada em fevereiro de 2023 e iniciada em junho do mesmo ano. O objetivo foi estimar o diferencial por raça/cor da pele no acesso à cirurgia de reconstrução mamária após a mastectomia. A abordagem permitiu analisar as diferenças de tendência (inclinação) das taxas mensais entre os dois grupos de mulheres antes e depois da intervenção. 

Participantes 

O estudo considerou as cirurgias de mastectomias e reconstruções mamárias realizadas pelo sexo feminino entre janeiro de 2010 e dezembro de 2024. A unidade de análise foram as taxas mensais por grupo racial. O número de cirurgias foi obtido a partir da variável *proc_id*. Os códigos para as mastectomias foram selecionados com base em Silva et al.^21^, e os códigos para os procedimentos de reconstrução mamária foram obtidos conforme Almeida et al.^11^. 

Variáveis do estudo 

Foram analisados os marcadores de raça/cor da pele e regiões de residência. A variável *raca_cor* identificou a raça/cor da pele da usuária conforme estabelecido pelo Instituto Brasileiro de Geografia e Estatística, em que 1 indica raça/cor da pele branca, 2 preta, 3 parda, 4 amarela, 5 indígena, 99 ignorado ou "sem informação". As indicações 99 foram retiradas do estudo. No total, 120.492 cirurgias não tinham a informação do quesito raça/cor da pele, sendo 88.170 mastectomias e 32.322 cirurgias de reconstrução mamária. As mulheres indígenas foram retiradas da análise por conta da baixa representatividade na amostra (137 mastectomias e 23 reconstruções). 

A informação de raça/cor da pele se tornou obrigatória no Sistema de Informações Hospitalares em novembro de 2022, em que a opção "sem informação" para raça/cor da pele se tornou indisponível durante o preenchimento das informações. Nos dados, foi possível verificar que, durante o biênio de 2023 e 2024, não houve dados sem informação da raça/cor da pele. Esse registro é realizado pelos profissionais de saúde, logo não é uma autodeclaração. 

A análise se direcionou a dois grupos de mulheres: aquelas com indicação 2 e 3 na base de dados, ou seja, preta e parda (ambas classificadas como negras neste estudo); e as mulheres brancas e amarelas com códigos 1 e 4, respectivamente. A população negra reúne as pessoas autodeclaradas como pretas e pardas. Embora corresponda a uma ampla gama de fenótipos e diversas ancestralidades genômicas, tal categorização política e social é frequentemente adotada no país. 

A região de residência foi obtida por meio da variável *munic_res* que indica o código do município da usuária, em que o primeiro caractere igual a 1 corresponde à região Norte, 2 ao Nordeste, 3 ao Sudeste, 4 ao Sul e 5 ao Centro-Oeste. 

Fonte de dados/mensuração 

A base de dados foi extraída do Sistema de Informação Hospitalares em março de 2025 e é composta por dois arquivos disponíveis para download na Plataforma Transferência de Arquivos^20^. Neste estudo, foi utilizada a base reduzida, em que foi possível obter informações sobre as cirurgias e os quesitos raça/cor da pele e região de residência das usuárias. Os arquivos são mensais e organizados por estado da Federação. 

O indicador de reconstrução mamária (variável dependente) foi calculado como a taxa entre as reconstruções e as mastectomias, ou seja, em que *Taxa*_*it*_ é a relação entre cirurgias de reconstrução mamária e de mastectomias realizadas no grupo *i* (negras ou brancas e amarelas) durante o mês *t*. *Reconstruções*_*it*_ e *Mastectomias*_*it*_ indicam o número total de reconstruções e mastectomias do grupo *i* durante o mês *t*, respectivamente.

$$Taxa_{it}=\frac{Reconstruções_{it}}{Mastectomias_{it}}\cdot 100\left(1\right)$$

A usuária tem o direito de realizar a reconstrução imediatamente após a retirada do tumor, se houver as condições clínicas necessárias, ou assim que apresentar os requisitos^13^. Há três possibilidades: a reconstrução está indicada imediatamente após a mastectomia; a reconstrução não está indicada; ou, primeiramente, a usuária deve realizar o tratamento complementar com quimioterapia/radioterapia e, depois, a reconstrução da mama^5^. Em geral, a decisão sobre o melhor tratamento é personalizada conforme a especificidade do tumor, a faixa etária e as atividades cotidianas. Além disso, é compartilhada entre o mastologista, o cirurgião plástico, o oncologista e a usuária^5^. 

Os microdados não possuem a identificação das usuárias para acompanhar quando cada uma realizou a reconstrução. As taxas foram calculadas em cada mês para cada um dos dois grupos de mulheres. Isso implica que este estudo enriqueceu à literatura ao analisar o diferencial por raça/cor da pele com o uso de taxas mensais. 

O número anual de reconstruções mamárias não refletiu a quantidade de mulheres submetidas à mastectomia após o diagnóstico de câncer de mama^9^. A expressão (1) descreveu a capacidade mensal do SUS em oferecer o tratamento completo da doença. 

Viés 

A exclusão dos registros sem informação sobre raça/cor da pele atingiu parte substancial da amostra. Além disso, a pandemia de covid-19 reduziu as cirurgias eletivas entre 2020 e 2021. 

Tamanho do estudo 

Após a exclusão dos registros sem informação de raça/cor da pele e indígenas, a amostra final analisada foi composta por 160.290 reconstruções e 404.944 mastectomias. Por se tratar de dados administrativos populacionais, não foi realizado cálculo amostral. 

Métodos estatísticos 

O método denominado por séries temporais interrompidas com dados em painel foi descrito em Linden^22^. Explorou-se a intervenção da Portaria nº 127/2023 para obter o diferencial por raça/cor da pele na relação entre as cirurgias de reconstrução mamária e mastectomias no SUS. A Portaria estabeleceu estratégia excepcional para ampliar o acesso à reconstrução^14^ e foi incluída no Sistema de Gerenciamento da Tabela de Procedimentos, Medicamentos e Órteses, Próteses e Meios Auxiliares de Locomoção do SUS, popularmente conhecida como Tabela SUS em junho de 2023. 

A hipótese de identificação é que a mudança no nível ou na tendência das taxas é a mesma para o grupo de controle (brancas e amarelas) e para o grupo de tratamento (negras) se não tivesse ocorrido a intervenção^22^. A intervenção causada pela pandemia de covid-19 não é adequada nessa análise, pois nesse período foram interrompidas muitas cirurgias oncológicas. Consequentemente, isso reduziu o diferencial entre os grupos. O efeito da pandemia é sempre positivo ao se comparar as taxas com a redução substancial ocorrida a partir de março de 2020. 

O modelo foi estimado por mínimos quadrados ordinários, conforme a seguir. 

*Taxa*_*it*_ *= ß*_*0*_ *+ ß*_*1*_ *Tempo*_*it*_ *+ß*_*2*_ *Intervenção*_*it*_ *+ ß*_*3*_ *Tempo*_*it*_ *· Intervenção*_*it*_ *+ ß*_*4*_ *Negras*_*i*_ *+ ß*_*5*_ *Negras*_*i*_ *· Tempo*_*it*_ *+ ß*_*6*_ *Negras*_*i*_ *· Intervenção*_*it*_ *+ ß*_*7*_ *Negras*_*i*_ *· Intervenção*_*it*_ *· Tempo*_*it*_ *+ ε*_*it*_ (2)

em que: 

*Taxa*_*it*_ é a variável dependente calculada em (1); 

*Tempo*_*it*_ é uma variável contínua que varia de 1 a 180, representando janeiro de 2010 a dezembro de 2024 para cada grupo *i*; 

*Intervenção*_*it*_ é uma variável binária que recebe 1 a partir de junho de 2023; 

*Tempo*_*it*_ *· Intervenção*_*it*_ é uma variável contínua que indica o tempo decorrido desde a intervenção, ou seja, em julho de 2023 é 1, em agosto de 2023 é 2 e assim por diante; 

*Negras*_*i*_ é uma variável binária que indica 1 para o grupo de mulheres negras e 0 para as mulheres brancas e amarelas; 

*Negras*_*i*_ *· Tempo*_*it*_ é o tempo decorrido desde o início do estudo para o grupo de mulheres negras; 

*Negras*_*i*_ *· Intervenção*_*it*_ é uma variável binária que recebe 1 para a intervenção para as mulheres negras; 

*Negras*_*i*_ *· Intervenção*_*it*_ *· Tempo*_*it*_ indica o tempo desde a intervenção para as mulheres negras; e

*ε*_*it*_ é o termo de erro. 

A equação (2) testou a comparabilidade entre grupos. Os *ß*_*4*_ e *ß*_*5*_ indicam se os grupos estão equilibrados tanto no nível quanto na trajetória da variável de resultado no período de pré-intervenção^22^. Os coeficientes *ß*_*0*_ a *ß*_*3*_ medem os efeitos para as mulheres brancas e amarelas, enquanto os parâmetros *ß*_*4*_ a *ß*_*7*_ computam os efeitos para as mulheres negras. Portanto, *ß*_*4*_ e *ß*_*6*_ são os parâmetros de interesse e estimam o diferencial entre os dois grupos de mulheres antes e depois da intervenção em junho de 2023, respectivamente. A especificação de (2) permite capturar os efeitos heterogêneos, ajustando as diferentes trajetórias e os níveis da variável dependente antes e depois da intervenção^22^. Além disso, é possível verificar a efetividade da Portaria em ampliar o acesso às reconstruções. 

Os coeficientes da equação (2) foram estimados no software estatístico R. Os erros-padrão são robustos à heterocedasticidade, calculados a partir do estimador de White, conforme implementado pelo pacote *sandwich*. Os intervalos de confiança foram construídos ao nível de 95%. 

## Resultados 

A amostra total contou com 565.234 cirurgias realizadas em mulheres, sendo 71,6% (404.494) mastectomias e 28,4% (160.290) reconstruções mamárias. Foram descritos os procedimentos cirúrgicos utilizados para mensurar a variável dependente do modelo (Tabela 1). No Sul, 91,2% das reconstruções foram realizadas em mulheres brancas, e no Sudeste, 59,6%; no Norte, 81,6% ocorreram em mulheres pardas, no Nordeste, 74,2%, e no Centro-Oeste, 61,6% (Tabela 2).

**Tabela 1:** Código, descrição, quantidade (n) e percentual (%) de mastectomias e reconstruções mamárias realizados no Sistema Único de Saúde. Brasil, 2010-2024

Código	Descrição	n (%)
	Mastectomias	404,944 (100.0)
0416120032	Mastectomia simples em oncologia	14,554 (3.6)
0410010111	Setorectomia/quadrantectomia	179,537 (44.3)
0416120024	Mastectomia radical com linfadenectomia axilar em oncologia	87,855 (21.7)
0410010120	Setorectomia/quadrantectomia com esvaziamento ganglionar	23,014 (5.7)
0416120059	Segmentectomia de mama em oncologia	77,870 (19.2)
0410010065	Mastectomia simples	9,747 (2.4)
0410010057	Mastectomia radical com linfadenectomia	12,367 (3.1)
	Reconstruções mamárias	160,290 (100.0)
0410010090	Plástica mamária reconstrutiva pós-mastectomia com implante de prótese	17,527 (10.9)
0410010073	Reconstrução de mama feminina não estética	79,646 (49.7)
0416080081	Reconstrução com retalho miocutâneo	60,658 (37.8)
0410010219	Reconstrução mamária pós-mastectomia total (procedimento criado pela Portaria nº 127/2023)	2,459 (1.5)

**Tabela 2:** Frequências absoluta (n) e relativa (%) das cirurgias de mastectomias e reconstruções mamárias realizadas por região de residência e raça/cor da pele. Brasil, 2010-2024

Cirurgia/mulheres	Brasil n (%)	Norte n (%)	Nordeste n (%)	Sudeste n (%)	Sul n (%)	Centro-Oeste n (%)
Mastectomias	404,944	22,092	98.181	189.267	74,989	20,415
Brancas	190,251 (47.0)	1,883 (8.5)	11,867 (12.1)	104,420 (55.2)	66,622 (88.8)	5,459 (26.7)
Pretas	25,015 (6.2)	413 (1.9)	5,998 (6.1)	15,147 (8.0)	2,693 (3.6)	764 (3.7)
Pardas	181,991 (44.9)	19,050 (86.2)	76,861 (78.3)	66,956 (35.4)	5,269 (7.0)	13,855 (67.9)
Amarelas	7,687 (1.9)	746 (3.4)	3,455 (3.5)	2,744 (1.4)	405 (0.5)	337 (1.7)
Reconstruções	160,290	3,636	41,715	77,041	30,845	7,053
Brancas	84,051 (52.4)	503 (13.8)	7,118 (17.1)	45,881 (59.6)	28,130 (91.2)	2,419 (34.3)
Pretas	8,346 (5.2)	72 (2.0)	2,532 (6.1)	4,780 (6.2)	788 (2.6)	174 (2.5)
Pardas	65,702 (41.0)	2,967 (81.6)	30,972 (74.2)	25,677 (33.3)	1,740 (5.6)	4,346 (61.6)
Amarelas	2,191 (1.4)	94 (2.6)	1,093 (2.6)	703 (0.9)	187 (0.6)	114 (1.6)

No período 2010-2024, a proporção de reconstruções em relação às mastectomias foi maior entre mulheres brancas e amarelas em comparação a mulheres negras em todos os anos analisados (Figuras 1 e 2). Em 2010, as proporções foram de 26,6% entre mulheres brancas e amarelas e 21,1% entre mulheres negras, diferença de 5,5 pontos percentuais. Em 2020, por conta da interrupção dos procedimentos eletivos, observou-se redução nas proporções em ambos os grupos, com valores de 38,0% entre mulheres brancas e amarelas e 34,4% entre mulheres negras, o que resultou na diferença de 3,6 pontos percentuais. Após esse período, as proporções voltaram a crescer e atingiram 69,9% e 53,9%, respectivamente, em 2024, mantendo a diferença de 16,0 pontos percentuais entre os grupos (Figuras 1 e 2).

As taxas mensais permitiram a estimação da equação (2) para o Brasil e macrorregiões de residência da usuária (Norte, Nordeste, Sudeste, Sul e Centro-Oeste) a partir de filtros na base de dados. Em alguns meses, as taxas mensais apresentaram proporções superiores a 100% ou não puderam ser calculadas nos períodos em que não foram realizadas cirurgias de reconstrução. Na região Norte, foram eliminadas 20 observações na amostra, sete meses tinham taxas acima de 100%, e, em treze meses, não ocorreram cirurgias de reconstrução. Tais problemas foram observados em maior número entre 2010 e 2014. As regiões com maior completude de informações foram o Sudeste e o Sul, com 360 e 359 observações (Tabela 3).

**Figura 1:** Comparação anual das taxas de reconstrução mamária por grupo de mulheres. Brasil, 2010-2024

**Figura 2:** Séries mensais das taxas de reconstrução mamária por grupo de mulheres. Brasil, 2010-2024

Os coeficientes significativos para as variáveis *Negras*_*i*_ e *Negras*_*i*_ *· Intervenção*_*it*_ da equação (2) apontaram a existência de diferencial por raça/cor da pele no acesso à reconstrução mamária no SUS (Tabela 3). O coeficiente *ß*_*4*_ da variável *Negras*_*i*_ representou o grupo de mulheres negras na equação (2) antes da intervenção e apresentou valor negativo e significativo nas estimações, exceto para o Centro-Oeste (Tabela 3). Isso indicou que as mulheres negras apresentaram taxas da relação de cirurgia de reconstrução mamária e mastectomias menores em comparação ao grupo de mulheres brancas e amarelas.

**Tabela 3:** Diferença absoluta (em pontos percentuais) nas taxas de reconstrução mamária entre mulheres negras e mulheres brancas/amarelas antes e depois da intervenção da Portaria nº 127/2023, intervalos de confiança de 95% (IC95%) e observações mensais por região de residência. Brasil, 2010-2024

Região	Antes da intervenção (IC95%)	Depois da intervenção (IC95%)	Meses
Brasil	-12.9 (-16.8; -8.9)	-28.6 (-44.1; -13.0)	351
Norte	-15.4 (-20.9; -10.8)	1.4 (-13.1; 16.0)	340
Nordeste	-11.7 (-15.8; -7.5)	-21.0 (-46.3; 4.5)	340
Sudeste	-8.0 (-11.0; -5.0)	-0.4 (-10.6; 9.7)	360
Sul	-6.9 (-10.8; -3.0)	-10.9 (-24.4; 2.5)	359
Centro-Oeste	1.8 (-3.8; 7.4)	-8.7 (-28.1; 10.7)	352

Para o Brasil, a diferença entre os dois grupos foi 12,9 pontos percentuais (intervalo de confiança de 95%, IC95% -16,8; -8,9) a favor das mulheres brancas e amarelas no período pré-intervenção (Tabela 3). Nas regiões, o diferencial foi 15,4 pontos percentuais (IC95% -20,9; -10,8) no Norte, 11,7 pontos percentuais (IC95% -15,8; -7,5) no Nordeste, 8,0 pontos percentuais (IC95% -11,0; -5,0) no Sudeste e 6,9 pontos percentuais (IC95% -10,8; -3,0) no Sul. Os modelos das regiões Nordeste e Sul obtiveram os maiores coeficientes de determinação, enquanto os do Norte e do Centro-Oeste obtiveram os menores (Tabela 3).

Nas estimativas pós-intervenção, o coeficiente *ß*_*6*_ da interação *Negras*_*i*_ *· Intervenção*_*it*_ da equação (2) foi negativo e altamente significativo apenas para o Brasil. O diferencial aumentou para 28,6 pontos percentuais (IC95% -44,1; -13,0) a favor das mulheres brancas e amarelas (Tabela 3). 

## Discussão

Estes resultados indicaram que existiu diferencial por raça/cor da pele na relação entre reconstrução mamária e mastectomia entre janeiro de 2010 e dezembro de 2024. As taxas foram mais elevadas para mulheres brancas e amarelas em comparação com mulheres negras. No Brasil, até a intervenção da Portaria nº 127/2023, o diferencial era 12,9 pontos percentuais e se ampliou para 28,6 pontos percentuais a partir de junho de 2023. Além disso, os efeitos foram heterogêneos entre as regiões. Os maiores diferenciais foram observados no Norte e no Nordeste. Apenas no Centro-Oeste, o coeficiente que indica o diferencial não foi significativo.

A quantidade de reconstruções não se equiparou à de mastectomias^9^^-^^12^. Utilizando-se de diferentes recortes anuais, foram verificadas as proporções de reconstruções sobre mastectomias realizadas por ano^9^^-^^12^. Dessa maneira, este estudo contribuiu com a literatura ao analisar a influência da raça/cor da pele e ao verificar a heterogeneidade regional com o uso do método de séries temporais.

As limitações deste estudo foram reconhecidas. Primeiramente, estes resultados foram condicionados à precisão e à qualidade dos dados. Não foi possível garantir que não houve falhas no registro ou na omissão de informações. A informação de raça/cor da pele se tornou obrigatória nos registros do Sistema de Informações Hospitalares a partir de novembro de 2022. Diante disso, as análises podem apresentar algumas distorções. Em segundo lugar, não foi possível controlar os efeitos da pandemia de covid-19 nas estimativas. No período mais restritivo, foi observada redução nas cirurgias oncológicas^23^, sendo assim qualquer comparação posterior ao período da pandemia terá o efeito positivo. Por último, as evidências do estudo não foram embasadas por modelo teórico que explique as relações entre todos os possíveis fatores envolvidos.

Estudos como este enfatizam a importância de bases de dados com informações completas para o quesito raça/cor da pele, visando delinear políticas para reduzir as desigualdades em saúde. Os resultados mostraram que o aumento de reconstruções, ao longo dos anos, não assegurou a redução das diferenças de acesso. Isso indicou a necessidade de políticas públicas equitativas para ampliar o acesso à cirurgia reparadora.

A reconstrução mamária é essencial para a recuperação física e emocional das mulheres que vivenciam o tratamento do câncer^8^. O número anual de reconstruções mamárias não refletiu o aumento de mulheres com diagnóstico de câncer de mama submetidas à mastectomia^9^. Portanto, iniciativas como a Portaria nº 127/2023, a qual viabilizou que hospitais de referência estejam aptos a realizar a reconstrução mamária pós-mastectomia^14^, são importantes para ampliar o acesso ao tratamento completo^9^ e o bem-estar das usuárias.

Estes achados revelaram o diferencial por raça/cor da pele no acesso à reconstrução mamária e estão de acordo com a literatura. Muitos estudos sobre câncer de mama ressaltam as desigualdades em saúde enfrentadas pelas mulheres negras. Elas possuem menor acesso ao rastreamento^16^^,^^24^, apresentam estadiamento mais avançado^17^ e maior risco de mortalidade^19^. As interações desses múltiplos fatores podem influenciar as diferenças no acesso à reconstrução, visto que casos mais graves de câncer podem apresentar mais contraindicações ao procedimento. Tais resultados adversos também são observados em indicadores socioeconômicos. As mulheres negras participam menos do mercado de trabalho, são mais afetadas pela pobreza e concluem menos o ensino superior^25^.

As diferenças regionais também se destacaram. Norte e Nordeste foram as regiões que apresentaram os maiores diferenciais no acesso à reconstrução e foram as localidades com maior proporção de população preta e parda (79% e 73%, respectivamente)^26^. Apesar dos avanços nas últimas décadas, desigualdades persistem. Na região Norte, o câncer de mama é o segundo mais incidente, atrás do câncer de colo do útero^27^, e apresenta baixas taxas de rastreamento^28^, maior tempo de espera para cirurgia de câncer de mama^29^ e acesso limitado ao diagnóstico e ao tratamento oncológico devido à vasta extensão territorial^30^.

O conceito de interseccionalidade é útil para explicar essas desigualdades, pois se refere à compreensão de que características como raça/cor da pele, classe social, gênero e local de residência moldam e reforçam desigualdades sociais complexas^15^. Portanto, há diferencial por raça/cor da pele nas taxas mensais entre as cirurgias de reconstrução mamária e mastectomias, e o efeito é heterogêneo entre as regiões. Esse resultado é decorrente das diferenças em muitos indicadores sociais que afetam o acesso ao tratamento completo do câncer de mama. 

## Data Availability

O banco de dados utilizado na pesquisa está disponível em: https://doi.org/10.48331/scielodata.NPPFOF.
